# Long-term outcome in young women with breast cancer: a population-based study

**DOI:** 10.1007/s10549-016-3983-9

**Published:** 2016-09-13

**Authors:** Hanna Fredholm, Kristina Magnusson, Linda S. Lindström, Hans Garmo, Sonja Eaker Fält, Henrik Lindman, Jonas Bergh, Lars Holmberg, Fredrik Pontén, Jan Frisell, Irma Fredriksson

**Affiliations:** 1 Department of Molecular Medicine and Surgery, Karolinska Institutet, Stockholm, Sweden; 2 Department of Breast- and Endocrine Surgery, Karolinska University Hospital, 171 76 Stockholm, Sweden; 3 Science for Life Laboratory, Department of Immunology, Genetics and Pathology, Uppsala University, Uppsala, Sweden; 4 Department of Biosciences and Nutrition, Karolinska Institutet, Stockholm, Sweden; 5 Department of Surgical Sciences, Uppsala University Hospital, Regional Cancer Center, Uppsala University, Uppsala, Sweden; 6 Division of Cancer Studies, Faculty of Life Sciences and Medicine, King’s College London, London, UK; 7 Department of Radiology, Oncology and Radiation Science, Uppsala University Hospital, Uppsala University, Uppsala, Sweden; 8 Department of Oncology and Pathology, Cancer Center Karolinska and Karolinska Oncology, Radiumhemmet, Karolinska University Hospital, Karolinska Institutet, Stockholm, Sweden

**Keywords:** Breast cancer, Young age, Subtype, Luminal B, Early stage, Prognosis, Population-based

## Abstract

**Purpose:**

Whether young age at diagnosis of breast cancer is an independent risk factor for death remains controversial, and the question whether young age should be considered in treatment decisions is still to be answered.

**Methods:**

From a population-based cohort of 22,017 women with breast cancer, all women <35 years (*n* = 471) were compared to a random sample of 700 women aged 35–69 years from the same cohort. Information on patient and tumor characteristics, treatment, and follow-up was collected from the medical records. Tissue microarrays were produced for analysis of classical biomarkers. Breast cancer-specific survival (BCSS), distant disease-free survival (DDFS), and locoregional recurrence-free survival (LRFS) by age were compared using women 50–69 years as reference.

**Results:**

At 10 years follow-up, women <35 years and 35–39 years had a worse BCSS [age <35 years 69 % (HR 2.75, 95 % CI 1.93–3.94), age 35–39 years 76 % (HR 2.33, 95 % CI 1.54–3.52), age 40–49 years 84 % (HR 1.53, 95 % CI 0.97–2.39), and age 50–69 years 89 % (reference)]. The worse BCSS was statistically significant in stages I–IIa and Luminal B tumors. At multivariate analysis age <35 years and 35–39 years confined a risk in LRFS (HR 2.13, 95 % CI 1.21–3.76 and HR 1.97, 95 % CI 1.06–3.68) but not in DDFS and BCSS. In the subgroup of women <40 years with luminal tumors stage I–IIa, low age remained an independent risk factor also in DDFS (HR 1.87, 95 % CI 1.03–3.44).

**Conclusion:**

Young women have a high risk of systemic disease even when diagnosed in an early stage. The excess risk of relapse is most pronounced in Luminal B tumors, where low age is an independent prognostic factor of DDFS and LRFS.

**Electronic supplementary material:**

The online version of this article (doi:10.1007/s10549-016-3983-9) contains supplementary material, which is available to authorized users.

## Introduction

Young women with breast cancer have a worse prognosis than middle-aged women [1–7], partly explained by diagnosis at a later stage [2–4, 6, 8] and by a higher proportion of unfavorable tumor characteristics. Young women more often have high grade, hormone receptor-negative, Her2-positive tumors, and also more often multifocality, high proliferation, and lymphovascular invasion (LVI) [2, 3, 6, 9–13]. Young women have a higher proportion of intrinsic breast cancer subtypes [14] associated with a worse prognosis: the triple-negative, Her2-positive, and Luminal B subtypes [5, 13, 15–18]. Recently, the prognostic significance of young age has been shown to differ between the different subtypes. Whether young age is an independent prognostic marker for poorer survival even when taking subtype into account remains controversial [5, 10, 15, 18].

In a previous large registry-based cohort study, we found women <35 years to have a worse survival than middle-aged women [3]. Stage at diagnosis was a major explanatory factor; however, the excess risk of breast cancer death seen in younger women was only present in early disease, most pronounced in women with small tumors. After correction for stage and tumor characteristics, young age remained an independent risk factor for death.

As it is not likely that young age in itself confers a worse prognosis, but rather this reflects other associations we had not been able to correct for in our registry-based study, we continued with in-depth studies on a large subpopulation from the original cohort. We collected detailed data from the medical records (tumor characteristics, heredity, parity, and treatment), re-evaluated slides (grade, LVI), and collected tumor tissue for TMA providing us with an immunohistochemical (IHC) surrogate of the intrinsic breast subtypes for this population-based cohort study with almost complete and long-term follow-up to study the independent effect of young age on breast cancer-specific survival (BCSS), distant disease-free survival (DDFS), and locoregional recurrence-free survival (LRFS).

## Methods

### Study design

Through the regional breast cancer registries in two of Sweden’s six health-care regions, a population-based cohort of 22,017 women with a primary invasive breast cancer diagnosed from 1992 to 2005 at 69 years of age or younger were identified. All women <35 years at diagnosis (*n* = 471) were compared to random sampled groups of women aged 35–39 years (*n* = 200), 40–49 years (*n* = 200), and 50–69 years (*n* = 300) (Flow chart Fig. S1). The sample size was set after power calculations based on the effect sizes from the registry-based study [3]. To reach a power of 80 % at a 95 % significance level, we needed 326 individuals to detect a difference in BCSS and 262 individuals to detect a difference in LRFS.

Information on patient and tumor characteristics, including the treatments given and follow-up until the end of 2012 or until death was collected from the medical records. For women with synchronous bilateral breast cancer, the largest tumor was chosen as the index cancer. Staging was performed using the American Joint Committee on Cancer, 7th edition [19]. The study conforms to the STROBE and REMARK guidelines [20, 21].

### Tumor material

Archival haematoxylin and eosin stained sections and corresponding formalin-fixed and paraffin-embedded tumor blocks were retrieved and histologically reviewed for grade [22] and LVI. Re-sectioning and re-staining were carried out when archival sections were missing. When histological review was not possible, data on grade and LVI were extracted from pathology reports. The presence of multifocality (defined as two or more invasive tumor foci separated by at least 1 cm) and ductal carcinoma in situ (DCIS) with extensive growth (defined as >25 % of the tumor consisting of DCIS with intraductal component also beyond the edge of the invasive tumor) was extracted from the original pathology reports.

TMAs were generated for protein expression profiling using IHC. TMA production, IHC staining, slide scanning, and evaluation of outcome were performed in accordance with strategies and standards used in the Human Protein Atlas project [23, 24]. All patients with tumor material available, 983/1120 (88 %), were included in the set of TMAs. For IHC, the following primary antibodies were used: ER (estrogen receptor) 1:150 (M7047, Dako, Glostrup, Denmark), PR (progesterone receptor) 1:1000 (M3569, Dako), Ki67 1:200 (M7240, Dako), and Her2 1:1000 (A0485, Dako). IHC was performed as previously described [25]. In brief, 4 µm sections of the TMA blocks were cut and automated IHC was done using a Lab Vision Autostainer 480 (Thermo Fisher Scientific). The IHC-stained and mounted TMA slides were scanned at ×20 magnification with a ScanScope XT system (Aperio Technologies, Vista, USA). The high-resolution digital images of each tissue core were annotated with respect to the outcome of IHC staining. ER was defined as positive when >1 % of the tumor cell nuclei were positive and PR as positive when >25 % of the tumor cell nuclei were positive. Ki67 was considered high when >20 % of the tumor cell nuclei were positive [26, 27]. Her2 was annotated using Her2 ASCO guidelines [28]. Membrane staining intensity of 3+ was considered positive, while 2+ was further verified through chromogenic in situ hybridization (CISH) to determine Her2-gene amplification [28–30]. CISH was performed on an automated Ventana BenchMark ULTRA IHC/ISH Staining Module (Ventana Medical Systems, Inc Tuscon, AZ, USA) using the INFORM HER2 Dual ISH DNA Probe Cocktail. CISH-stained slides were examined under the microscope and the amount of positive Her2 signals scored in tumor cell nuclei. The outcome was scored as Her2 amplified (>6 dots or clusters of positive signal) or non-Her2 amplified (≤6 dots per nuclei).

To define the intrinsic breast cancer subtypes, we used surrogate definitions based on central IHC re-evaluation of ER, PR, Ki67, and Her2 according to the St Gallen consensus statement [27]. Luminal A was defined as ER+, PR+, Her2−, and Ki67 low, Luminal B as ER+, PR+, Her2−, and Ki67 high or ER+, PR−, Her2−, and any Ki67, Luminal-Her2 as ER+ and Her2+, any PR or Ki67, Her2-positive (non-luminal) as ER−, PR− and Her2+, any Ki67 and triple-negative as ER−, PR− and Her2−, any Ki67.

### Statistical analysis

Endpoints were BCSS, DDFS, and LRFS. BCSS was calculated using time from diagnosis to death from breast cancer censoring for end of follow-up. DDFS was estimated using time from diagnosis to distant recurrence or death from breast cancer, whichever came first censoring for the end of follow-up. LRFS was calculated using time from diagnosis to locoregional recurrence as first event. Kaplan–Meier curves were used to estimate survival time [31] as death from other causes than breast cancer was uncommon in this population. Survival curves were compared using log-rank test [32]. Cox proportional-hazards models were used to estimate the univariate and multivariate hazard ratios (HR) and 95 % confidence intervals (95 % CI) [33]. All statistical tests were two-sided and *p* values < 0.05 were deemed significant. All calculations were performed using IBM SPSS Statistics v22.0 (SPSS Inc. Illinois, USA).

## Results

### Population characteristics

Data on patient and tumor characteristics divided by age group are shown in Table 1. Women <35 years had larger tumors and more often involved lymph nodes than women aged 50–69 years. Fewer women <35 years presented with stage I disease. Women <35 years more often had tumors that were grade III, hormone receptor negative, Her2-positive, and high Ki67. This translates to a lower proportion of the luminal subtypes and a higher proportion of the triple-negative and Her2-positive subtypes among younger women. Multifocal disease, LVI, and the presence of extensive DCIS were more common in women <35 years. Altogether, characteristics in women 35–39 years were similar to those in women <35 years, whereas the characteristics in women 40–49 years group together well with those aged 50–69 years.

**Table 1:** Patient- and tumor characteristics for women with primary breast cancer stage I–III diagnosed 1992–2005, by age at diagnosis (*N* = 1120)

	<35 years		35–39 years		40–49 years		50–69 years	
	*n* = 445		*n* = 190		*n* = 192		*n* = 293	
	No.	(%)	No.	(%)	No.	(%)	No.	(%)
Year of diagnosis								
1992–1997	169	(38.0)	82	(43.2)	86	(44.8)	89	(30.4)
1998–2002	175	(39.3)	61	(32.1)	62	(32.3)	132	(45.1)
2003–2005	101	(22.7)	47	(24.7)	44	(22.9)	72	(24.6)
Detection by screening	6	(1.3)	5	(2.6)	45	(23.4)	167	(57.0)
Heredity^a^								
Any heredity	187	(42.0)	73	(38.4)	59	(30.7)	80	(27.3)
≥1 first grade relative	81	(18.2)	37	(19.5)	25	(13.0)	50	(17.1)
Tumor size								
1–10 mm	67	(15.1)	27	(14.2)	35	(18.2)	77	(26.3)
11–20 mm	148	(33.3)	72	(37.9)	80	(41.7)	136	(46.4)
21–50 mm	189	(42.5)	72	(37.9)	70	(36.5)	67	(22.9)
>51 mm	38	(8.5)	16	(8.4)	6	(3.1)	11	(3.8)
Missing	3	(0.7)	3	(1.6)	1	(0.5)	2	(0.7)
Lymph node status								
Node neg	227	(51.0)	90	(47.4)	116	(60.4)	214	(73.0)
1–3 nodes pos	126	(28.3)	68	(35.8)	47	(24.5)	57	(19.5)
>4 nodes pos	92	(20.7)	32	(16.8)	29	(15.1)	22	(7.5)
Stage								
I	14	(32.4)	63	(33.2)	87	(45.3)	172	(58.7)
IIa	126	(28.3)	50	(26.3)	44	(22.9)	74	(25.3)
IIb	71	(16.0)	35	(18.4)	28	(14.6)	21	(7.2)
III	103	(23.1)	40	(21.1)	33	(17.2)	26	(8.9)
Unstaged	1	(0.2)	2	(1.1)	0		0	
Grade (Elston)								
I	21	(5.3)	21	(12.7)	31	(17.9)	75	(27.3)
II	140	(35.6)	63	(38.2)	79	(45.7)	131	(47.6)
III	232	(59.0)	81	(49.1)	63	(36.4)	69	(25.1)
Missing	52		25		19		18	
Estrogen receptor^b^								
Pos	208	(47.2)	122	(64.9)	146	(77.7)	225	(78.1)
Neg	233	(52.8)	66	(35.1)	42	(22.3)	63	(21.9)
Missing	4		2		4		5	
Progesterone receptor^b^								
Pos	155	(35.4)	85	(45.2)	114	(60.6)	145	(51.1)
Neg	283	(64.6)	103	(54.8)	74	(39.4)	139	(48.9)
Missing	7		2		4		9	
Ki-67 (%)								
Low ≤20	70	(18.8)	42	(26.9)	67	(40.1)	127	(51.2)
High >20	302	(81.2)	114	(73.1)	100	(59.9)	121	(48.8)
Missing	73		34		25		45	
Her2								
Neg	296	(79.6)	127	(81.4)	150	(90.4)	225	(91.8)
Pos	76	(20.4)	29	(18.6)	16	(9.6)	20	(8.2)
Missing	73		34		26		48	
Subtype								
Luminal A	27	(7.7)	23	(15.1)	40	(25.8)	59	(25.9)
Luminal B	132	(37.5)	66	(43.4)	80	(51.6)	117	(51.3)
Luminal-Her2	35	(9.9)	16	(10.5)	7	(4.5)	10	(4.4)
Her2-positive	40	(11.4)	13	(8.6)	8	(5.2)	9	(3.9)
Triple-negative	118	(33.5)	34	(22.4)	20	(12.9)	33	(14.5)
Unclassified	93		38		37		65	
Presence of:								
LVI^b^	139	(31.2)	43	(22.6)	39	(20.3)	32	(10.9)
Invasive multifocality	96	(21.6)	39	(20.5)	35	(18.2)	46	(15.7)
Extensive DCIS	92	(20.7)	43	(22.6)	30	(15.6)	37	(12.6)

^a^Any family history of breast or ovarian cancer

^b^Data retrieved by re-evaluation with IHC (ER and PR) or reviewed by a pathologist (LVI). If missing data, information was retrieved from medical records

Treatment was performed according to the national guidelines for each time period, closely following international practice. Data on treatment by age group are shown in Table 2 and time trends of systemic treatment in relation to age, tumor size, lymph node status, grade, and subtype are presented in Fig. S2.

**Table 2:** Given treatment for women with primary breast cancer stage I–III diagnosed 1992–2005, by age at diagnosis (*N* = 1120)

	<35 years		35–39 years		40–49 years		50–69 years	
	*n* = 445		*n* = 190		*n* = 192		*n* = 293	
	No.	(%)	No.	(%)	No.	(%)	No.	(%)
Breast surgery								
BCS	206	(46.3)	94	(49.5)	117	(60.9)	194	(66.2)
Mastectomy	239	(53.7)	94	(49.5)	74	(38.5)	99	(33.8)
No surgery	0		2	(1.1)	1	(0.5)	0	
Chemotherapy								
No	109	(24.5)	48	(25.3)	103	(53.6)	204	(69.6)
Yes	336	(75.5)	142	(74.7)	89	(46.4)	89	(30.4)
CMF	78	(23.2)	45	(31.7)	27	(30.3)	24	(27.0)
FEC	208	(61.9)	82	(57.7)	54	(60.7)	60	(67.4)
Taxanes	47	(14.0)	15	(10.6)	6	(6.7)	5	(5.6)
Other	3	(0.9)	0		2	(2.2)	0	
Proportion neoadjuvant	76	(17.1)	29	(15.3)	13	(6.8)	8	(2.7)
Chemotherapy when N+	214	(98.2)	95	(95.0)	72	(94.7)	61	(77.2)
Chemotherapy when hormone rec pos_a_	169	(67.6)	85	(65.9)	65	(41.7)	59	(25.0)
Trastuzumab								
Yes	18	(4.0)	5	(2.6)	3	(1.6)	4	(1.4)
No	427	(96.0)	185	(97.4)	189	(98.4)	289	(98.6)
Radiotherapy								
Yes	358	(80.4)	149	(78.4)	160	(83.3)	231	(78.8)
No	87	(19.6)	41	(21.6)	32	(16.7)	62	(21.2)
Breast radiation when BCS	196	(95.1)	92	(97.9)	113	(96.6)	187	(96.4)
Chest wall radiation when mastectomy	162	(67.8)	57	(60.6)	46	(62.2)	43	(43.4)
Axillary radiation when N+	168	(77.1)	68	(68.0)	49	(64.5)	58	(73.4)
Endocrine therapy								
Yes	208	(46.7)	90	(47.4)	109	(56.8)	190	(64.8)
No	237	(53.3)	100	(52.6)	82	(42.7)	102	(34.8)
Missing	0		0		1	(0.5)	1	(0.3)
Endocrine therapy^b^ when hormone rec pos^a^	176	(70.4)	80	(62.0)	97	(62.2)	177	(75.0)
Ovarian suppression when hormone rec pos^a^	79	(31.9)	28	(22.0)	17	(11.1)	4	(1.7)

*BCS* breast conserving surgery, *CMF* cyclophosphamide, methotrexate, 5-fluorouracil, *FEC* 5-fluorouracil, epirubicin, cyclophosphamide, *N+* lymph node positive

^a^Hormone receptor positive defined as either ER pos or PR pos

^b^Endocrine therapy including ovarian suppression

Median follow-up time was 10 years (range 0–20). In the group aged <35 years, 90 of 445 had a locoregional recurrence as first event. The corresponding figures were for women 35–39 years 37 of 190, 40–49 years 27 of 192, and 50–69 years 22 of 293. Distant disease occurred in 169 of 445 women <35 years, in 59 of 190 women aged 35–39 years, in 47 of 192 women aged 40–49 years, and in 42 of 293 women aged 50–69 years.

### Univariate analysis

Univariate analyses of risk factors for breast cancer death stratified by age are shown in Table 3. The increased risk of breast cancer death in young versus middle-aged women was significant during the earlier part of the studied period and mainly noted in tumors with favorable characteristics, namely: small tumor size, low grade, Her2-negativity, and no LVI.

**Table 3:** Univariate analysis of risk factors for breast cancer death by age for women with stage I–III breast cancer diagnosed 1992–2005 (*N* = 1120)

	<35 years		35–39 years		40–49 years		50–69 years
	*n* = 445		*n* = 190		*n* = 192		*n* = 293
Unadjusted	**2.75**	**(1.93**–**3.94)**	**2.33**	**(1.54**–**3.52)**	1.53	(0.97–2.39)	
Year of diagnosis							
1992–1997	**2.18**	**(1.31**–**3.63)**	**2.04**	**(1.16**–**3.59)**	1.28	(0.70–2.35)	
1998–2002	**4.02**	**(2.16**–**7.50)**	**2.93**	**(1.37**–**6.27)**	1.80	(0.78–4.16)	
2003–2005	1.90	(0.79–4.54)	1.33	(0.45–3.95)	1.17	(0.37–3.68)	
Non screening detection	**1.80**	**(1.17**–**2.78)**	1.54	(0.95–2.49)	1.16	(0.69–1.96)	
Positive heredity	1.47	(0.84–2.58)	1.41	(0.73–2.73)	1.15	(0.56–2.35)	
Tumor size (mm)							
≤20	**3.42**	**(1.91**–**6.12)**	**3.19**	**(1.65**–**6.20)**	1.75	(0.85–3.63)	
21–50	1.60	(0.93–2.76)	1.49	(0.79–2.78)	1.24	(0.65–2.37)	
≥51	1.26	(0.51–3.11)	0.55	(0.18–1.70)	0.23	(0.03–1.93)	
Lymph node status							
Negative	**2.10**	**(1.22**–**3.62)**	**2.30**	**(1.21**–**4.39)**	0.63	(0.26–1.49)	
1–3 nodes positive	**2.63**	**(1.33**–**5.19)**	1.90	(0.90–4.00)	1.20	(0.50–2.89)	
≥4 nodes positive	1.41	(0.70–2.87)	1.05	(0.45–2.41)	2.05	(0.94–4.47)	
Stage							
I	**3.07**	**(1.41**–**6.68)**	**3.75**	**(1.58**–**8.90)**	1.24	(0.44–3.48)	
IIa	1.55	(0.82–2.94)	1.24	(0.56–2.78)	0.24	(0.05–1.06)	
IIb	1.71	(0.71–4.08)	1.20	(0.45–3.15)	0.83	(0.29–2.39)	
III	1.42	(0.73–2.80)	1.04	(0.48–2.27)	1.94	(0.92–4.10)	
Grade							
I–II	**3.25**	**(1.81**–**5.81)**	**2.38**	**(1.18**–**4.81)**	1.64	(0.80–3.36)	
III	1.46	(0.87–2.47)	1.37	(0.75–2.51)	0.91	(0.46–1.83)	
Estrogen receptor							
Positive	**2.89**	**(1.85**–**4.53)**	**2.28**	**(1.36**–**3.85)**	1.36	(0.78–2.39)	
Negative	**1.91**	**(1.03**–**3.52)**	1.85	(0.91–3.73)	1.59	(0.73–3.49)	
Progesterone receptor							1.00 (ref.)
Positive	**2.77**	**(1.53**–**5.01)**	**2.45**	**(1.26**–**4.79)**	1.10	(0.52–2.31)	
Negative	**2.37**	**(1.51**–**3.73)**	**2.04**	**(1.20**–**3.48)**	**1.91**	**(1.07**–**3.41)**	
Ki67 (%)							
Low ≤20	**3.15**	**(1.49**–**6.67)**	1.33	(0.46–3.82)	1.72	(0.73–4.06)	
High ≥21	**1.70**	**(1.08**–**2.68)**	1.65	(0.98–2.78)	1.09	(0.61–1.96)	
Her2							
Negative	**2.36**	**(1.55**–**3.60)**	**2.15**	**(1.31**–**3.53)**	1.27	(0.74–2.17)	
Positive	1.45	(0.56–3.74)	0.78	(0.25–2.46)	1.38	(0.42–4.52)	
Subtype							
Luminal A	1.84	(0.49–6.86)	0.55	(0.06–4.67)	0.29	(0.03–2.44)	
Luminal B	**2.30**	**(1.27**–**4.19)**	**2.30**	**(1.18**–**4.49)**	1.64	(0.82–3.28)	
Luminal-Her2	0.84	(0.23–3.02)	0.75	(0.18–3.16)	0.75	(0.13–4.50)	
Her2-positive	1.77	(0.41–7.67)	0.56	(0.08–3.98)	2.20	(0.40–12.03)	
Triple-negative	1.26	(0.61–2.61)	1.35	(0.57–3.21)	1.09	(0.39–3.07)	
Lymphovascular invasion							
No	**2.66**	**(1.74**–**4.06)**	**2.12**	**(1.29**–**3.49)**	1.14	(0.64–2.01)	
Yes	1.56	(0.77–3.15)	1.57	(0.71–3.47)	1.60	(0.71–3.60)	
Invasive multifocality							
No	**3.08**	**(2.04**–**4.63)**	**2.18**	**(1.33**–**3.55)**	1.62	(0.97–2.71)	
Yes	1.76	(0.84–3.71)	**2.26**	**(1.00**–**5.12)**	1.12	(0.43–2.91)	
Extensive DCIS							
No	**3.82**	**(1.93**–**7.54)**	**4.03**	**(1.89**–**8.62)**	1.93	(0.85–4.41)	
Yes	**5.11**	**(1.20**–**21.74)**	**5.30**	**(1.19**–**23.68)**	4.63	(0.96–22.27)	
Locoregional recurrence^a^							
No	**2.75**	**(1.82**–**4.17)**	**2.11**	**(1.28**–**3.48)**	1.53	(0.91–2.59)	
Yes	1.44	(0.71–2.94)	1.50	(0.69–3.27)	0.96	(0.40–2.31)	

Hazard ratio (95 % confidence interval) for risk of breast cancer death according to age and one additional risk factor

Bold values indicate statistical significance at the *p* < 0.05 level

^a^Locoregional recurrence as first event

**Fig. 1:**
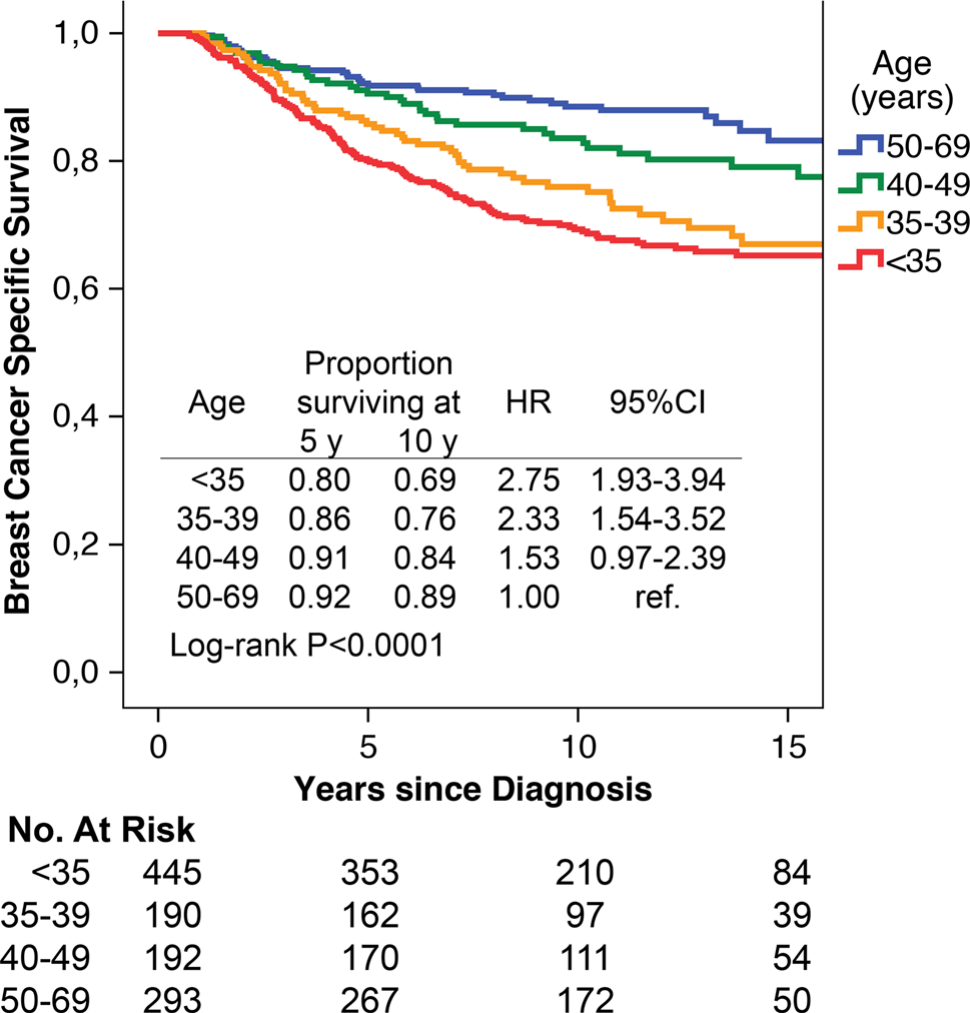
Breast cancer-specific survival by age in a population-based cohort of 1120 women with primary breast cancer stage I–III diagnosed 1992–2005 divided by age <35 years, 35–39 years, 40–49 years, and 50–69 years. Proportion of women surviving at 5, 10, and 15 years from diagnosis. Hazard ratios (HR) of breast cancer death are given with their 95 % confidence intervals (95 % CI). Survival curves are compared by log-rank test

At 10-year follow-up, the BCSS was for women <35 years 69 % (HR 2.75, 95 % CI 1.93–3.94), for women 35–39 years 76 % (HR 2.33, 95 % CI 1.54–3.52), for women 40–49 years 84 % (HR 1.53, 95 % CI 0.97–2.39), and women 50–69 years 89 % (HR = 1.00 reference) (Fig. 1).

**Fig. 2:**
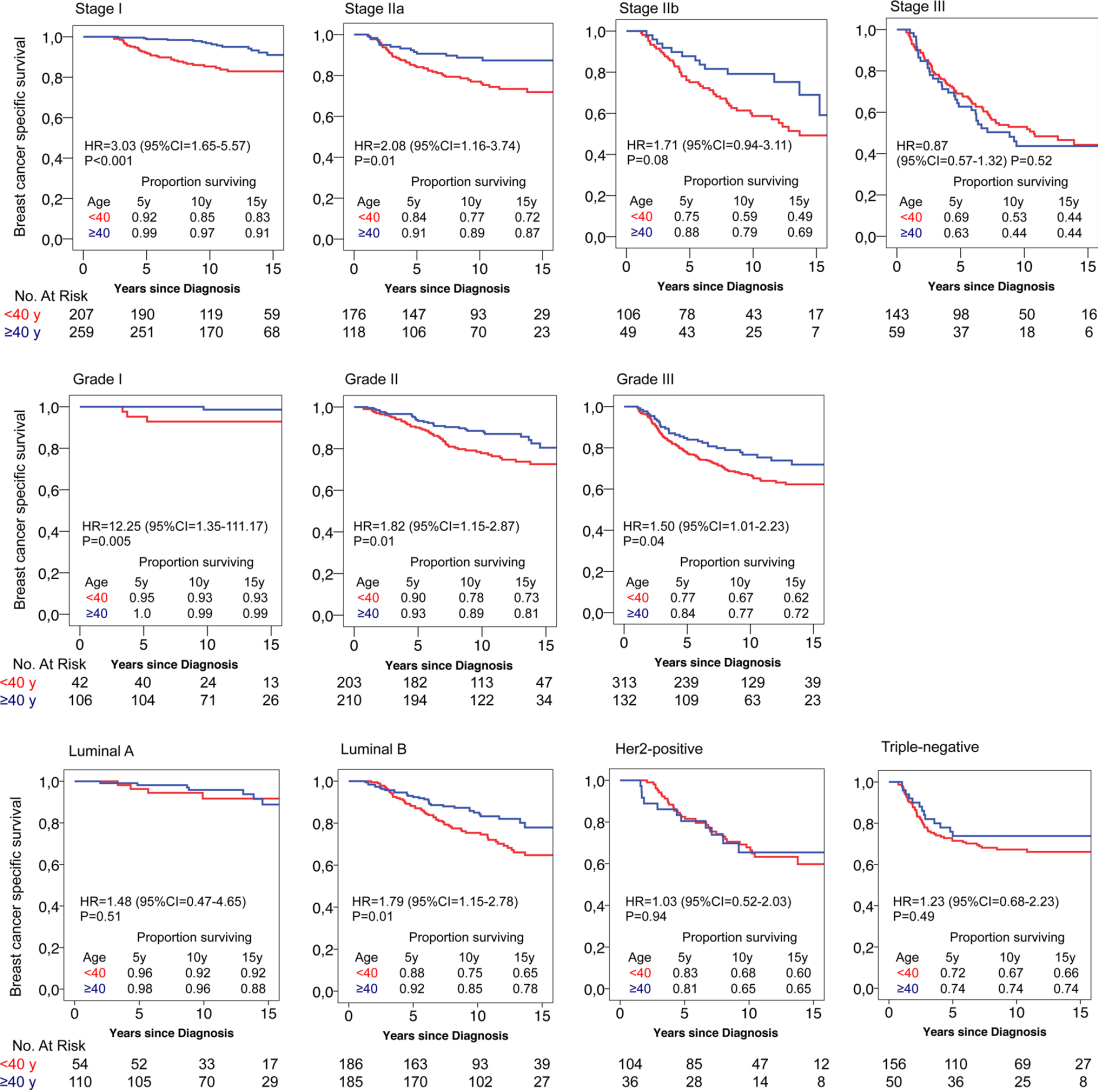
Breast cancer-specific survival by age, stage, grade, and subtype for women with primary breast cancer stage I-III diagnosed 1992–2005 (*N* = 1120) divided by age <40 years and ≥40 years. Hazard ratios (HR) are given with their 95 % confidence intervals (95 % CI). Survival curves are compared by log-rank test. Proportion of women surviving at 5, 10, and 15 years from diagnosis

Figure 2 shows BCSS by tumor characteristics and age. Women aged <40 years had a statistically significantly worse survival than women ≥40 years in stages I and IIa (HR 3.03, 95 % CI 1.65–5.57 and HR 2.08, 95 % CI 1.16–3.74), irrespective of tumor grade (grade I; HR 12.25, 95 % CI 1.35–111.17, grade II; HR 1.82, 95 % CI 1.15–2.87 and grade III; HR 1.50, 95 % CI 1.01–2.23), and in the Luminal B subtype (HR = 1.79, 95 % CI = 1.15-2.78). In women <40 years, the best survival was seen in those with Luminal A tumors (10-year BCSS 92 %) while it was markedly worse in the other subtypes (Luminal B 75 %, Her2-positive 68 % (in this analysis Luminal-Her2 and Her2-positive combined), and triple-negative 67 %).

### Multivariate analysis

In the multivariate analysis (Table 4), successively correcting for year of diagnosis, stage at diagnosis, detection mode, grade, subtype, and systemic treatment, young age (<35 years and 35–39 years) was an independent risk factor in LRFS (HR 2.13, 95 % CI 1.21–3.76 and HR 1.97, 95 % CI 1.06–3.68) but not in DDFS or BCSS.

**Table 4:** Multivariate analysis of prognostic factors^a^ affecting breast cancer death, distant disease, and locoregional recurrence by age

	<35 years		35–39 years		40–49 years		50–69 years
	*N* = 445		*N* = 190		*N* = 192		*N* = 293
Breast cancer death							
Unadjusted	**2.75**	**(1.93**–**3.94)**	**2.33**	**(1.54**–**3.52)**	1.53	(0.97–2.39)	1.00 (ref)
+Year	**2.69**	**(1.88**–**3.85)**	**2.23**	**(1.48**–**3.38)**	1.45	(0.92–2.28)	
+Stage	**1.80**	**(1.25**–**2.60)**	1.42	(0.93–2.17)	1.13	(0.72–1.78)	
+Detection mode	1.39	(0.93–2.07)	1.10	(0.70–1.72)	0.94	(0.59–1.50)	
+Grade	1.16	(0.77–1.73)	0.94	(0.59–1.49)	0.85	(0.53–1.36)	
+Subtype	1.10	(0.73–1.64)	0.93	(0.59–1.47)	0.86	(0.53–1.38)	
+Systemic treatment	1.04	(0.68–1.58)	0.88	(0.55–1.41)	0.84	(0.52–1.36)	
Distant disease							
Unadjusted	**3.11**	**(2.22**–**4.36)**	**2.37**	**(1.60**–**3.53)**	**1.74**	**(1.15**–**2.64)**	1.00 (ref)
+Year	**3.04**	**(2.17**–**4.26)**	**2.28**	**(1.53**–**3.39)**	**1.65**	**(1.09**–**2.50)**	
+Stage	**2.09**	**(1.48**–**2.96)**	1.46	(0.97–2.18)	1.29	(0.85–1.96)	
+Detection mode	**1.61**	**(1.10**–**2.35)**	1.13	(0.73–1.73)	1.07	(0.70–1.66)	
+Grade	1.41	(0.96–2.06)	1.02	(0.66–1.57)	1.00	(0.65–1.55)	
+Subtype	1.40	(0.96–2.05)	1.01	(0.65–1.55)	1.00	(0.65–1.55)	
+Systemic treatment	1.36	(0.91–2.02)	0.97	(0.62–1.52)	0.99	(0.64–1.54)	
Locoregional recurrence							
Unadjusted	**3.16**	**(1.98**–**5.04)**	**2.88**	**(1.70**–**4.89)**	**1.94**	**(1.11**–**3.41)**	1.00 (ref)
+Year	**3.09**	**(1.94**–**4.94)**	**2.80**	**(1.65**–**4.78)**	**1.85**	**(1.05**–**3.25)**	
+Stage	**2.88**	**(1.79**–**4.64)**	**2.60**	**(1.52**–**4.45)**	**1.78**	**(1.01**–**3.15)**	
+Detection mode	**2.38**	**(1.37**–**4.12)**	**2.15**	**(1.18**–**3.93)**	1.58	(0.87–2.86)	
+Grade	**2.11**	**(1.21**–**3.67)**	**1.96**	**(1.07**–**3.59)**	1.50	(0.82–2.72)	
+Subtype	**2.09**	**(1.20**–**3.65)**	**1.94**	**(1.06**–**3.57)**	1.51	(0.83–2.74)	
+Systemic treatment	**2.13**	**(1.21**–**3.76)**	**1.97**	**(1.06**–**3.68)**	1.51	(0.83–2.75)	

Women with stage I–III breast cancer diagnosed 1992–2005 (*N* = 1120). Women age 50–69 serves as reference category. Hazard ratio (95 % confidence interval)

Bold values indicate statistical significance at the *p* < 0.05 level

^a^Adjusted for year of diagnosis (1992–1997, 1998–2002, 2003–2005), stage (*tumor size* 1–10, 11–20, ≥20 mm, missing and *lymph node status*; node neg, node pos), detection mode (screening or clinically detected), grade (Elston I, II, III, missing), subtype (Lum A, Lum B, Lum-Her2, Her2-pos, Triple-neg, unclassified), systemic treatment (chemotherapy and endocrine treatment including ovarian suppression)

**Fig. 3:**
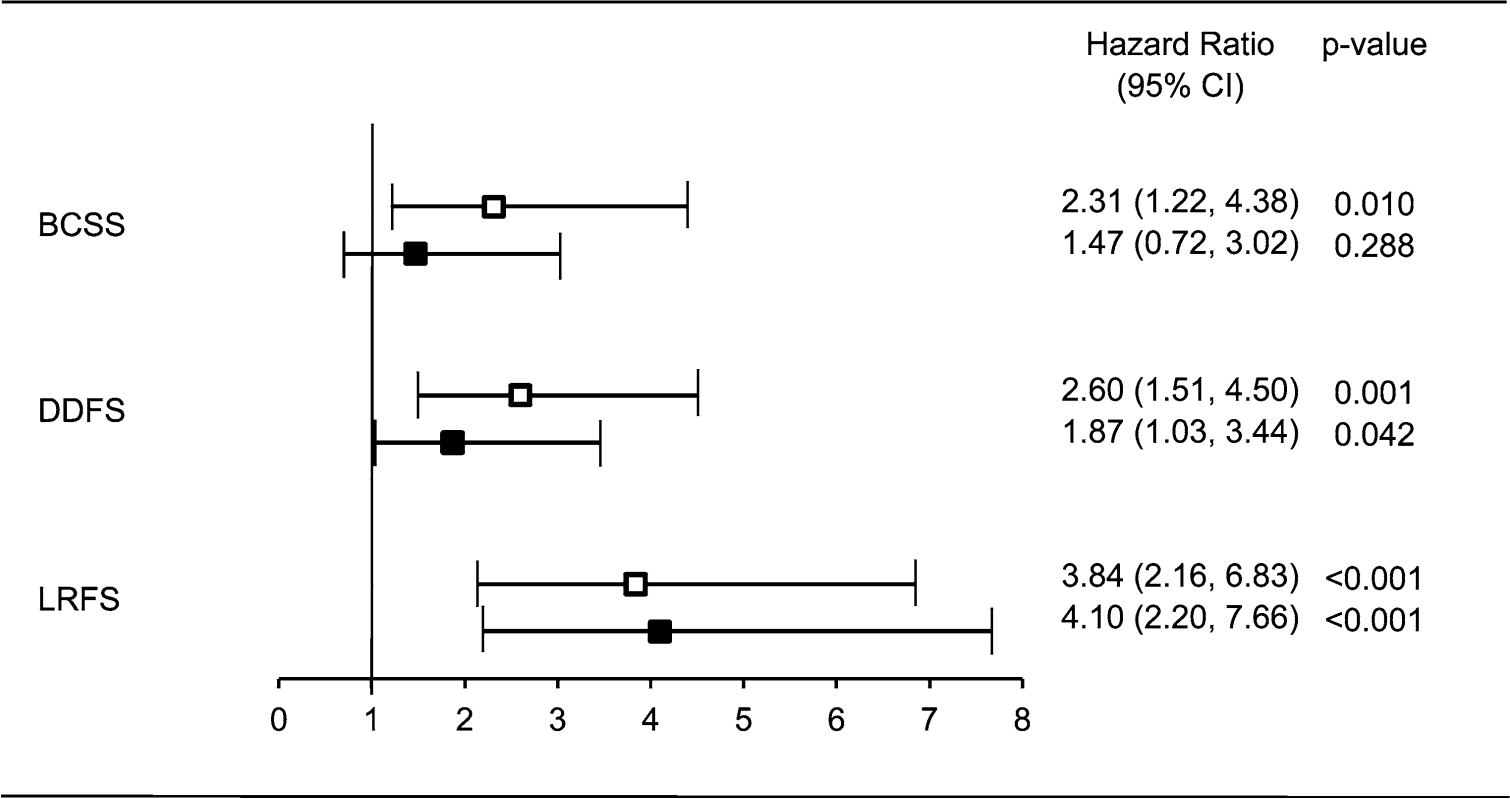
Forest plot of multivariate Cox regression of risk of event for women with stage I–IIa, estrogen receptor positive, and Her2-negative breast cancer (*N* = 389) by age <40 years (*n* = 152) versus reference ≥40 years (*n* = 237). *BCSS* Breast cancer-specific survival, *DDFS* distant disease-free survival, *LRFS* locoregional recurrence-free survival. *Open square* Crude, *filled square* adjusted for diagnostic period, tumor size, lymph node status, grade, subtype (Luminal A or Luminal B), endocrine therapy, and chemotherapy

To focus on the subpopulation of women where the survival analyses indicated substantial differences between women aged <40 and ≥40 years (Luminal Her-2 negative breast cancer stage I-IIa), we performed a separate multivariate analysis (Fig. 3). Age <40 years was a statistically significant independent risk factor in DDFS (HR 1.87, 95 % CI 1.03–3.44) and in LRFS (HR 4.10, 95 % CI 2.20–7.66), but not in BCSS (HR 1.47, 95 % CI 0.72–3.02).

## Discussion

This population-based cohort study included 1120 women with breast cancer stage I–III of which 445 were <35 years at diagnosis with a median follow-up of 10 years. Women aged <35 years and 35–39 years had more advanced stage at diagnosis and a higher proportion of Her2-positive and triple-negative subtypes and less common Luminal A subtype. Women <35 years and 35–39 years received more intense treatment reflecting their stage and subtype distribution. Women <40 years had a worse BCSS compared to women ≥40 years in stage I and IIa, in all tumor grades and in the Luminal B subtype. At multivariate analysis, age remained an independent risk factor in LRFS but not significantly in BCSS or DDFS. In women with luminal early-stage disease, young age was an independent risk factor also of DDFS.

Treatment was given according to national guidelines and best international practice at that time. The number of women <35 years and 35–39 years is large, with detailed data on patient, tumor, and treatment characteristics and follow-up extracted from medical records. The long-term follow-up is nearing completion.

In a central pathology review, we re-evaluated grade and LVI and re-analyzed prognostic markers with modern methods at one single laboratory. Using IHC methods to separate Luminal A from Luminal B tumors has limitations [34]. In this study, we performed new IHC-analyses on archival material to avoid the effects of low intra- and inter-laboratory reproducibility and different antibodies for testing. To validate our results with regard to the arbitrarily set cutoffs, we performed a sensitivity analysis using alternative subtype definitions; grade instead of Ki67, Ki67 cutoff 14 %, ER-positive cutoff >10 % stained nuclei, which did not change the results.

During the study period of 14 years, treatment regimes have changed, and the time trends have not been the same in the compared age groups. More intense treatment was offered to young women and modern regimes were introduced earlier, which might have led to an underestimation of age-related survival differences in the multivariate analyses.

Many studies have shown young women with breast cancer to have a worse prognosis compared to their older counterparts. Our findings demonstrate that the differences in BCSS between age groups diminished over time, and lost significance during the last part of the studied period. In a recent Canadian study, outcomes for young breast cancer patients across two time periods were compared to determine whether the poor prognosis persists in the context of modern adjuvant therapies. There was an improvement in breast cancer outcome over time for all subgroups, but age <40 continued to predict inferior survival despite modern therapies [18]. Published data from The Surveillance, Epidemiology, and End Results program showed improved outcomes for young women with breast cancer over time, however restricted only to women with ER-positive disease [35].

The difference in prognosis between age groups has consistently been reported to be particularly evident in young women with ER-positive tumors [36–40]. More recently, the prognostic significance of young age has been shown to be most prominent in the Luminal B subtype [5, 12, 15, 18, 41] even though some reports have indicated an increased risk compared with older women also among young with triple-negative [15, 16] and Her2-positive subtypes [42, 43]. In the present study, women aged <40 had a significantly worse survival only in the Luminal B subtype. Thus, the effect of age seems to vary within tumor subtypes.

Morrison et al. found Luminal B tumors among young women to demonstrate more aggressive features, with significantly lower ER and PR levels, higher Ki67, and p53 overexpression, than in older women with the same subtype. The high proliferation and p53 level, coupled with low ER and PR expression in young women, suggests that these tumors may originate from less-differentiated luminal cells [13].

Using genomic expression analysis, Azim and colleagues could, even after adjustment for subtype, observe remaining genetic differences by age with enrichment of processes related to immature mammary epithelial cells, growth factor signaling, and down-regulation of apoptosis-related genes [5]. Johnson et al. studied age-related gene expression differences within and across breast cancer subtypes. After adjustment for subtype, four key genes for proliferation, invasion, and metastasis persisted, some of which predicted inferior disease-free survival in younger women [43]. Also Liao et al. demonstrated unique genomic signatures differentiating premenopausal breast cancer from postmenopausal breast cancer, with the differences being limited to ER-positive tumors [44].

Whether the age-related biological differences within subtypes fully can explain the worse outcome for young women, or if treatment also plays a major role here, remains unclear. Except for age-related differences in the given treatment, one must also consider age-related differences in compliance to and effect of treatment. In the present study, all women were undertreated by today’s standards, with chemotherapy given to only 76 % of women <35 years and endocrine treatment to those with hormone receptor-positive disease in only 70 %. Ovarian suppression was offered to one-third of the youngest women with hormone receptor-positive tumors. Some authors have found young women to be less compliant with endocrine treatment [45–47]. Women with Luminal B breast cancer derive less benefit from endocrine therapy compared to those with Luminal A breast cancer [48], and likewise less benefit from paclitaxel and doxorubicin-containing preoperative chemotherapy compared with HER2-enriched and basal-like breast cancers [49–51]. Studies on neoadjuvant chemotherapy in women with luminal tumors have shown women <40 years to have a higher rate of pathological complete response than women >50 years also with positive effect on survival [52]. However, survival differences between young and older women with luminal tumors have been demonstrated also in untreated cohorts [5, 39].

To conclude, the effect of age is modified by tumor subtype. Despite correction for biology and more intense treatment in the young, young age is an independent risk factor for systemic disease in women with early-stage luminal tumors, with a two-fold risk of distant disease. However, current prognostic markers cannot reliably discriminate the young women benefitting from more intense systemic therapy and studies on prognostic markers relevant in the young population, and especially for the Luminal B subtype, are urgently needed. Age remains an important variable in treatment decisions until new relevant predictive markers are found.

## Electronic supplementary material

Below is the link to the electronic supplementary material.

Fig. S1 Supplementary material 1 (PDF 72 kb)

Fig. S2 Supplementary material 2 (PDF 189 kb)
