# Supplementary figures and images for: Long-term outcome in young women with breast cancer: a population-based study
Source: Breast Cancer Res Treat. 2016 Sep 13;160(1):131–43. doi: 10.1007/s10549-016-3983-9 (PMC5050247; doi:10.1007/s10549-016-3983-9)

**Supplementary Figure S1. Patient flow diagram**

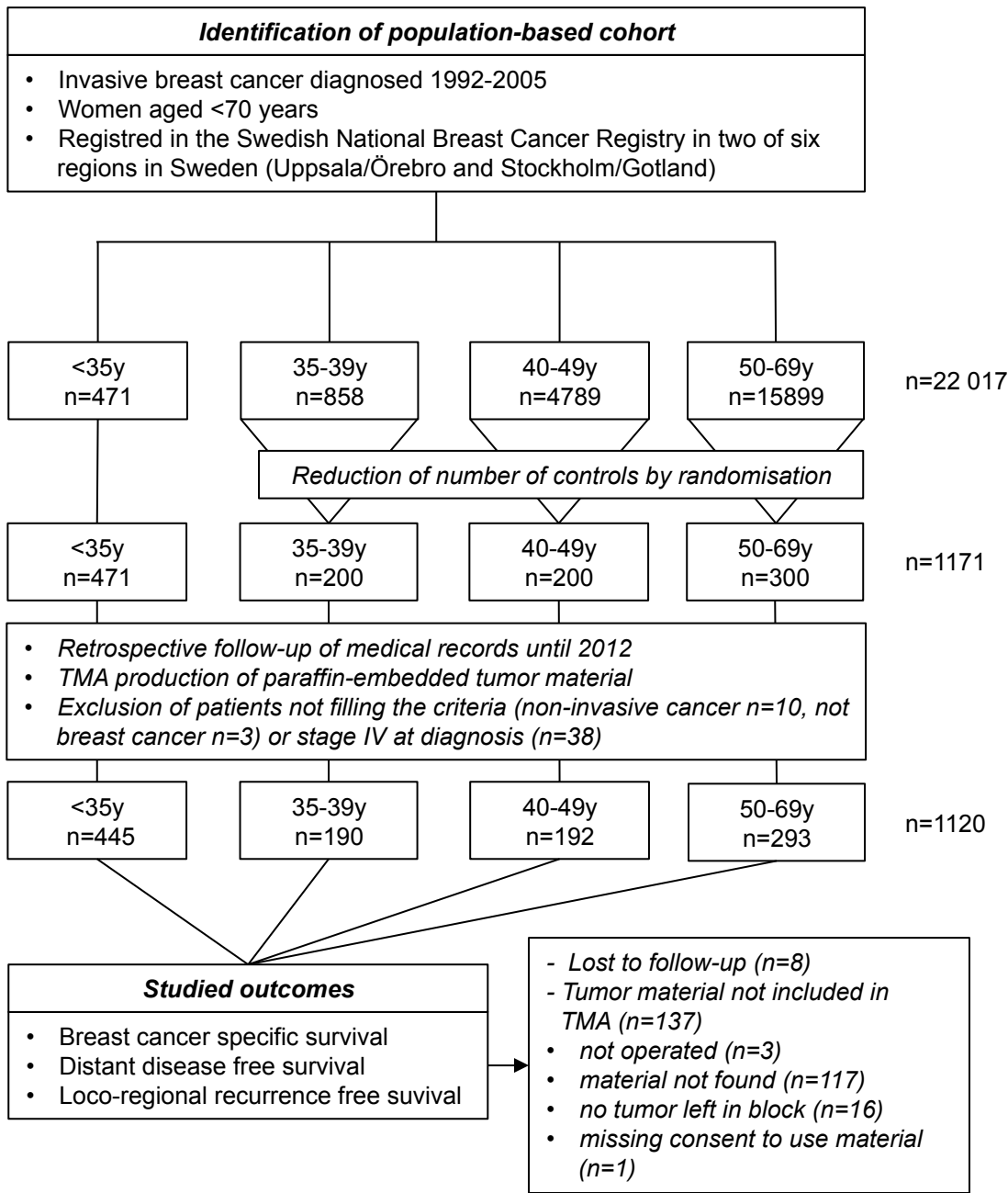

Supplement: Supplementary file 1 — Supplementary material 1 (PDF 72 kb) [file 10549_2016_3983_MOESM1_ESM.pdf]
