# Supplementary material for: Long-term outcome in young women with breast cancer: a population-based study
Source: Breast Cancer Res Treat. 2016 Sep 13;160(1):131–43. doi: 10.1007/s10549-016-3983-9 (PMC5050247; doi:10.1007/s10549-016-3983-9)
Supplement: Supplementary file 2 — Supplementary material 2 (PDF 189 kb) [file 10549_2016_3983_MOESM2_ESM.pdf]

Supplementary Figure S2. Time trends of systemic treatment in 1120 women with stage I-III breast cancer by tumor size, lymph node status, grade and subtype according to age (<40, n=635, ≥40, n=485)

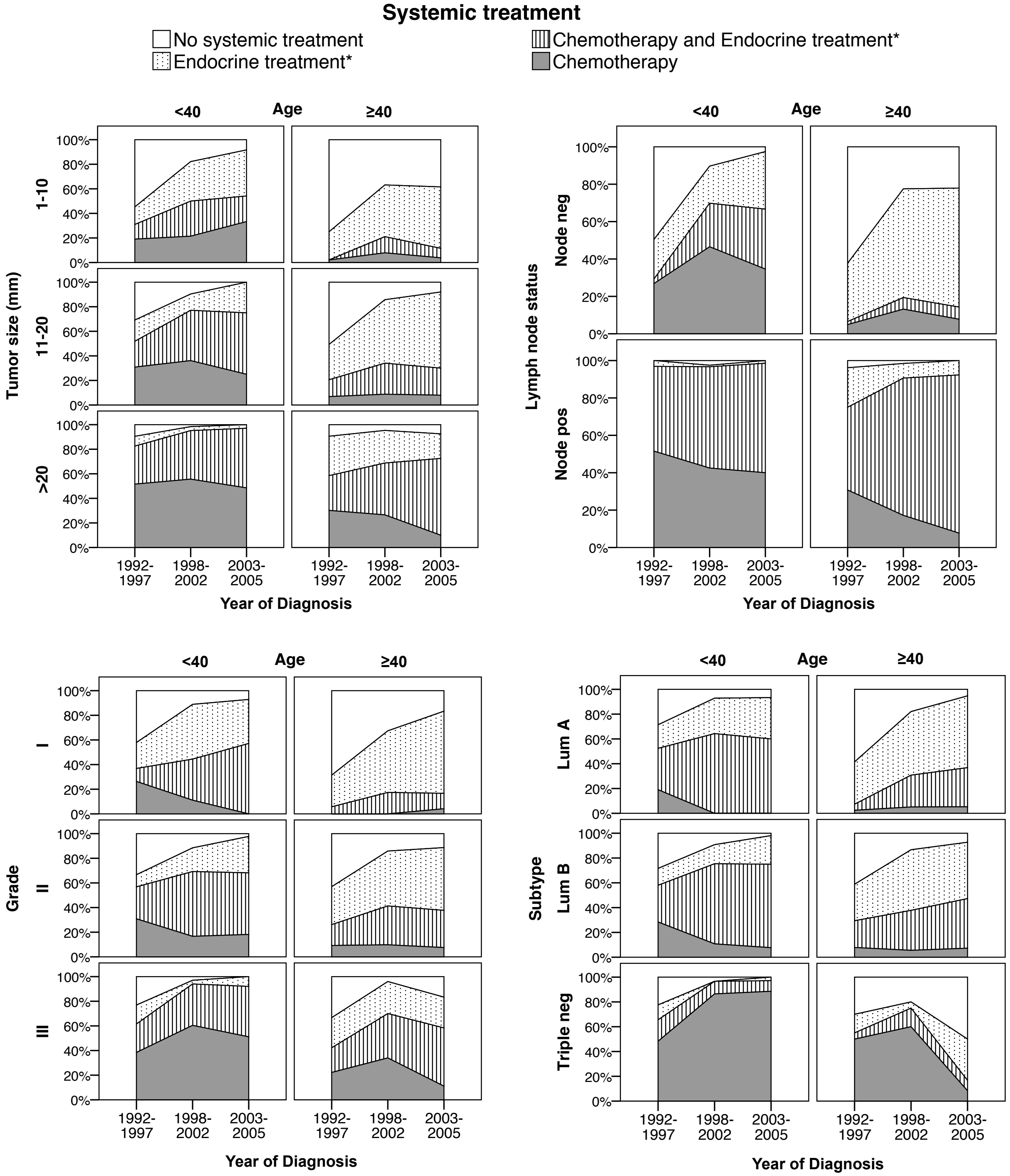

Un-informative cases are excluded from the analysis (systemic treatment; n=2, tumor size; n=3, grade; n=79). Only cases classified as subtype Luminal A, Luminal B and Triple-negative are included in the subtype panel (n=747). *Endocrine treatment including ovarian suppression.
